# *Bifidobacterium longum* can degrade the urinary *N* -glycoprotein Uromodulin.

**DOI:** 10.17912/micropub.biology.001724

**Published:** 2025-08-06

**Authors:** Elie Al Khoury, Jean-Philippe Gourdine

**Affiliations:** 1 Biochemistry & Molecular Biology, Lewis & Clark College, Portland, Oregon, United States of America; 2 Chemistry Department, Lewis & Clark College, Portland, Oregon, United States of America; 3 Biochemistry & Molecular Biology Program, Lewis & Clark College, Portland, Oregon, United States of America

## Abstract

Uromodulin (UMOD), the most abundant urinary glycoprotein, protects against urinary tract infections through glycan-mediated pathogen binding; nevertheless, its interactions with commensal bladder microbiota (urobiome) remain unexplored. Based on our previous bioinformatics analysis on the urobiome’s capacity to digest host glycans with glycosyl hydrolase genes (GHs), we hypothesized that UMOD may serve as a nutrient source for selected bacteria. We cultured one of them, *Bifidobacterium longum,* in a minimal medium supplemented solely with purified UMOD. Our results indicate that UMOD is degraded by *B. longum* , supporting a new role for UMOD in the urobiome’s metabolism.

**Figure 1. Probiotics B. longum subsp longum 35624 can digest purified UMOD:**
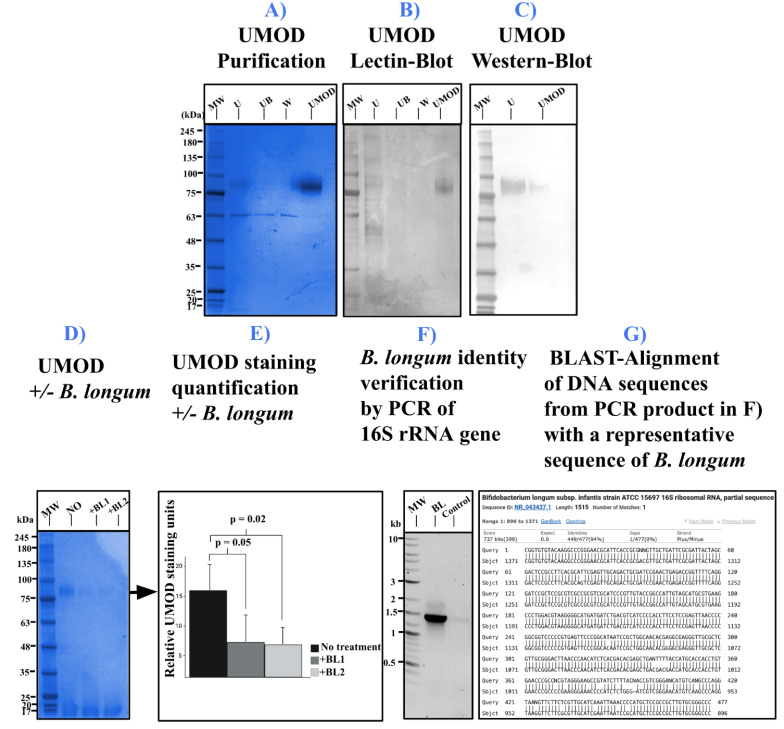
Electropherogram **(A)** and blots **(B-C)** of purified Uromodulin **.** **A)** Ammonium Bicarbonate precipitation of UMOD by SDS-PAGE revealed by Coomassie Blue **B)** Lectin-blot with Con A, and **C)** Immunoblot with mouse Anti-UMOD antibody followed by anti-mouse-Horseradish peroxidase. **D)** Supernatant of 0.22 µm filtered UMOD treated with MRS media without glucose alone (NO) or with *Bifidobacterium longum* after 16h (+ BL1 and + BL2) **E)** ImageJ quantification of UMOD staining (n=3) of **D** , p-value are statistically significant between control and *B. longum* (+BL1 and BL2) **F)** Electropherogram of PCR products on 1% Agarose; MW: Molecular Weight **G)** Exemplary of a sequence alignments with BLAST using DNA sequences from PCR products showed in **F** . *Legend: MW: Molecular Weight, U: Total Urine Fraction, W: Wash Fraction, FT: Flow Through, UMOD: Eluted UMOD. Black Arrow indicated the expected UMOD band. NO: No treatment; +BL1: with B. longum grown at OD _600nm_ = 0.15; +BL2: with B. longum grown at OD _600nm_ = 0.3; BL: B. longum; Control: No DNA.
*

## Description

Uromodulin (UMOD), also known as the Tamm-Horsfall glycoprotein, is the most abundant excreted urinary glycoprotein (~50 mg daily); it contains high amounts of *N* -glycans, averaging 30% of its mass (Li et al., 2021). UMOD is secreted by the kidneys and ends up in the bladder lumen, where it is involved in host defense against bacteria that cause urinary tract infections (UTIs) (Schaeffer et al., 2021). These host-pathogen interactions occur between UMOD’s high-mannose *N* -glycans and bacterial glycan-binding proteins, such as FimH of uropathogenic *Escherichia coli* (UPEC) (Weiss et al., 2020).

UMOD’s interactions with UPEC have been well studied (Pak et al., 2001; Raffi et al., 2005; Serafini-Cessi et al., 2005; Weiss et al., 2020), whereas its interactions with commensal bacteria that do not cause UTIs have not been explored yet. Indeed, urine possesses its microbiota in both healthy and diseased states (Hilt et al., 2014). Many urinary microbes possess enzymes that can digest host glycans (glycosaminoglycans, *N* -glycans, and *O* -glycans) with their specific glycoside hydrolases (GHs) and polysaccharide lyases (PLs) (Gourdine et al., 2025; Neugent et al., 2023). As UMOD is abundant and extensively *N* -glycosylated (Li et al., 2021; Lin et al., 2023; Weiss et al., 2020), it could potentially serve as a primary source of nutrients for bladder bacteria, as seen in other body sites such as the gut (Briliūtė et al., 2019; Garrido et al., 2012).

To test this hypothesis, we utilized a bacterium, *Bifidobacterium* *longum* , a member of both the healthy gut and bladder microbiota (Nickel et al., 2022), which possesses the GHs involved in *N* -glycans degradation (Cordeiro et al., 2019, 2023; Gourdine et al., 2025). As a proof-of-concept, we used an over-the-counter probiotic, *B.* *longum* *subsp longum* 35624, to test UMOD digestion. We first purified UMOD from voided urine samples from healthy female subjects (BIOIVT) using ammonium bicarbonate (ABC) precipitation (Li et al., 2021), followed by SDS-PAGE and staining with Coomassie Blue ( Fig. 1A ). We confirmed UMOD’s high-mannose *N* -glycosylation and identity, respectively, by Concanavalin A (ConA) Lectin-Blot ( Fig. 1B ) and by anti-UMOD immunoblot ( Fig. 1C ). We then cultured *B.* *longum* *subsp longum* 35624, in a defined minimal medium with UMOD as the sole nutrient source, and checked for UMOD degradation by SDS-PAGE of the supernatants following Garrido et al. (Garrido et al., 2012) ( Fig. 1D ). We quantified the decrease in UMOD staining using ImageJ ( Fig. 1E ). In parallel, we confirmed the identity of the commercially available *B. longum* by DNA extraction followed by PCR of the 16S rRNA gene and electrophoresis of the PCR products ( Fig. 1F ). PCR products were isolated and sequenced. DNA sequences were aligned using BLAST and the 16S ribosomal RNA sequences database from the National Center for Biotechnology Information (NCBI) (Boratyn et al., 2013) (Fig.1G).

Our results indicate:1) a high-yield purification of UMOD by ABC precipitation with proper high-mannose content ( Fig. 1A- C); 2) a decrease in UMOD staining by SDS-PAGE only in the presence of *B. longum* ( Fig. 1D- E), and 3) a genetic confirmation of the identity of an over-the-counter probiotic *B. longum* ( Fig. 1F- G). Overall, these results provide preliminary evidence for a novel role of UMOD in host–microbe interaction as a potential nutrient source for specific commensal bacteria. Our future experiments will involve culturing commensal bacteria isolated from the bladder with UMOD and an in-depth analysis of UMOD *N* -glycans digestion.

## Methods

*UMOD Purification*

UMOD was purified according to Li et al. (Li et al., 2021). Female urine samples acquired from BIOIVT were mixed with NaCl to achieve a final concentration of 0.58 M. Ammonium bicarbonate was added until a pH of 8 was reached. The resulting solution was stirred at 4℃ overnight, then centrifuged at 15,000 rpm for 20 minutes. The pellet was resuspended in 500 µL of deionized water and concentrated by centrifugation with a size exclusion filter (Millipore, molecular cut-off 50 kDa). UMOD purity was assessed by SDS-PAGE and stained with Coomassie Blue, or transferred onto nitrocellulose or polyvinylidene membranes. After blocking with Tris Buffer Saline with 0.1% Tween and 5% Bovine Serum Albumin or 1% Non-Fat dry Milk, membranes were incubated with biotinylated Concanavalin A (ConA; 1:1000), followed by Streptavidin-Horse Radish Peroxidase (HRP; Lectin-blot; 1:1000) or anti-UMOD (THP B2; 1:1000), followed by secondary antibody-HRP (m-IgG Fc BP-HRP; Immunoblot; 1:1000). Blots were revealed using a colorimetric method.

*Estimation of UMOD degradation*

One capsule of probiotic *B. longum* (~400 mg) was dissolved in 10 mL of Man-Rogosa-Sharpe Broth with 0.05 % thioglycolate (MRS-T) supplemented with 2% glucose filtered at 0.22 µm. The vial was incubated for 15 min at room temperature, then spun at minimal speed for 30 seconds. One volume of supernatant was mixed with one volume of MRS-TG for 48 hours at 37 °C and 250 rpm, until an optical density (OD _600 nm_ ) of 0.56 was reached. The culture was diluted and centrifuged at 13,000 rpm. Bacterial pellets were resuspended in 200 µL of MRS-T with 2.5 µg/µL of 0.22 µm-filtered UMOD. UMOD alone in 200 µL MRS-T was used as a negative control. Tubes were incubated for 14 hours at 37℃ without agitation, then centrifuged at 13,000 rpm for 1 minute. The supernatants were electrophorized (SDS-PAGE) as described previously. Coomassie blue-stained UMOD bands were quantified using ImageJ software (Schneider et al., 2012) in grayscale mode, with a box (x = 1008, y = 765) used to measure the mean pixel values. The same box size was used to select ten random sections of the electrophoregram picture without bands, and then averaged as background. Data were exported into a CSV file, which was then subtracted from 255 (n=3, for each bacterial dilution). Statistical analysis was performed between samples using Student's T-test.

*Validation of the probiotics identity*

*Bifidobacterium longum subsp longum* 35624 from Align Probiotics (Procter & Gamble), was acquired at the Albertson store (Lake Oswego, Oregon). One probiotics capsule was resuspended in 9 ml of sterile phosphate-buffered saline (PBS) and vortexed for 2 minutes (Lewis et al., 2016). One milliliter was centrifuged for 10 min at 7,500 rpm. DNA was extracted from the bacterial pellets using the QIagen DNeasy Blood & Tissue kit, and amplified by PCR using universal 16S rRNA primers 27F: AGAGTTTGATCCTGGCTCAG and 1492R: GGTTACCTTGTTACGACTT (Heuer et al., 1997). PCR products were electrophorised in 1% agarose gel in Tris Acetate Ethylene Diamine Tetraacetate (TAE) with SYBR Safe (Invitrogen), visualized under ultraviolet light (Biorad GelDoc). PCR products were extracted using a PCR Clean-up kit (Zymogen) and commercially sequenced (Sanger Sequencing, Azenta Genewiz). DNA sequences (Fasta) were compared to the NCBI database with BLAST (Boratyn et al., 2013).

## Reagents

**Table:** 

**Strain**	**Genotype**	**Available from**
Bifidobacterium longum subsp longum 35624	*Bifidobacterium longum*	Align Probiotics (Procter & Gamble)
**Lectin-Blot**	**Specificity**	**Description**
Concanavalin A (ConA-biotinylated)	α-D-mannose and α-D-glucose	Vector Laboratories
Horseradish Peroxidase Avidin D (Elisa Grade)	Biotin Secondary Antibody	Vector Laboratories
**Immunoblot**	**Animal & clonality**	**Description**
anti-UMOD (THP B-2)	anti-UMOD Mouse monoclonal IgG1	Santa Cruz Biotechnology
m-IgG Fc BP-HRP sc-525409	mouse anti-Fc HRP Conjugated	Santa Cruz Biotechnology
**Western Blot and Development**		**Company**
Tris-Glycine Running Buffer		Biorad
Non-fat dry milk	1% Blocking Solution	Kroger
Bovine Serum Albumin (BSA)	5% Blocking Solution	Sigma
Tween 20	polyoxyethylene sorbitan monolaureate	Biorad
Amersham Hybond-ECL Nitrocellulose Membrane		General Electric Healthcare
10x HRP Color Development Buffer		Biorad
HRP Color Reagent A		Biorad
HRP Color Reagent B		Biorad
**Reagent/Kits for Bacterial Analysis**	**Specificity**	**Available from**
DNA extraction kit	Blood and Soil	QIAgen
PCR kits	Taq PCR Kit E5000S	New England Biolabs.
PCR clean up	DNA Clean & Concentrator-25	Zymogen
100 bp DNA Ladder		New England Biolabs.
Agarose		Biorad
SybrSafe		Thermo
Tris Acetate EDTA (TAE)		10 x Biorad
MRS Broth		Cysteine 0.5 gCasein peptone, tryptic digest 10.00 gMeat extract 10.00 gYeast extract 5.00 gGlucose 20.00 gTween 80 1.00 gK2HPO4 2.00 gNa-acetate 5.00 g(NH4)3 citrate 2.00 gMgSO4 x 7 H2O 0.20 gMnSO4 x H2O 0.05 g0.5 g Sodium thioglycollateDistilled water 1000.00 mlAdjust pH to 6.2 - 6.5.Autoclave using liquid cycle.
Thioglycolate		Sigma
Glucose		Sigma
NaCl		Genesee Scientific
**SDS-PAGE**		**Company**
SurePAGE Bis-Tris 10x8	MOPS 4-12%; 12 wells	GenScript
BLUEstain Protein Ladder	11-245 kDa	GoldBio
Tris-MOPS SDS Running Buffer Powder		GenScript
Coomassie Blue	Coomassie 0.1%Ethanol 40%Acetate 10%Distilled water 49.9%	Sigma
**UMOD Purification**		**Company**
Ammonium Bicarbonate		Genesee Scientific
SpinFilter Centrifuge Tube	>50 kDa	Millipore

## Data Availability

Description: Partial DNA Sequences of 16S rRNA gene used for Fig 1G.. Resource Type: Dataset. DOI: https://doi.org/10.22002/hq8j1-nj790
